# Gene Mapping and Identification of a Missense Mutation in One Copy of *VRN-A1* Affects Heading Date Variation in Wheat

**DOI:** 10.3390/ijms24055008

**Published:** 2023-03-05

**Authors:** Qianwen Xue, Hongchun Xiong, Chunyun Zhou, Huijun Guo, Linshu Zhao, Yongdun Xie, Jiayu Gu, Shirong Zhao, Yuping Ding, Le Xu, Luxiang Liu

**Affiliations:** 1 College of Agriculture, Hubei Center for Collaborative Innovation of Grain Industry, Yangtze University, Jingzhou 434025, China; 2 National Engineering Laboratory for Crop Molecular Breeding, National Center of Space Mutagenesis for Crop Improvement, Institute of Crop Sciences, Chinese Academy of Agricultural Sciences, Beijing 100081, China

**Keywords:** heading date, wheat, *VRN-A1*, genetic mapping, copy number

## Abstract

Heading date (HD) is an important trait for wide adaptability and yield stability in wheat. The Vernalization 1 (*VRN1*) gene is a key regulatory factor controlling HD in wheat. The identification of allelic variations in *VRN1* is crucial for wheat improvement as climate change becomes more of a threat to agriculture. In this study, we identified an EMS-induced late-heading wheat mutant *je0155* and crossed it with wide-type (WT) Jing411 to construct an F_2_ population of 344 individuals. Through Bulk Segregant Analysis (BSA) of early and late-heading plants, we identified a Quantitative Trait Locus (QTL) for HD on chromosome 5A. Further genetic linkage analysis limited the QTL to a physical region of 0.8 Mb. Cloning and sequencing revealed three copies of *VRN-A1* in the WT and mutant lines; one copy contained a missense mutation of C changed to T in exon 4 and another copy contained a mutation in intron 5. Genotype and phenotype analysis of the segregation population validated that the mutations in *VRN-A1* contributed to the late HD phenotype in the mutant. Expression analysis of C- or T-type alleles in exon 4 of the WT and mutant lines indicated that this mutation led to lower expression of *VRN-A1*, which resulted in the late-heading of *je0155*. This study provides valuable information for the genetic regulation of HD and many important resources for HD refinement in wheat breeding programs.

## 1. Introduction

Wheat (*Triticum aestivum* L.) is the most widely grown staple crop worldwide. It contains a large and complex allohexaploid genome (2n = 6x = 42, AABBDD genome) [1]. Wheat cultivars are classified into winter and spring types based on whether it requires cold temperatures to promote flowering, and therefore can be well adaptable to different geographical conditions [2]. Heading date is an important factor that determines the ability of wheat to adapt to a wide range of different ecological environments, thus affecting global grain yield [3,4,5]. The identification of genes or alleles affecting heading time would facilitate the optimization of the heading stage by providing a target for marker-assisted selection in wheat breeding.

The main factors affecting heading time in wheat have been identified as the vernalization requirement and photoperiod [6,7]. Winter wheat can only transition from vegetative growth to reproductive growth after a long period of low-temperature induction [8,9]. *VRN1*, *VRN2*, and *VRN3* are the main regulatory factors affecting the vernalization time of wheat [10,11,12,13]. *VRN1*, encoding a MADS-box family protein, is the central genetic element in regulating vernalization. *VRN-A1*, *VRN-B1*, and *VRN-D1*, the three homologous genes of *VRN1*, are located on chromosomes 5A, 5B, and 5D, respectively [14,15,16]. The *VRN2* locus includes two tandem repeat genes, *ZCCT1* and *ZCCT2*, which encode the zinc finger and CCT domain-containing proteins [9]. Nucleotide deletion or mutation in the CCT domain was found to accelerate spring growth in diploid and tetraploid wheat varieties [17]. *VRN3* is an orthologous gene of the *Arabidopsis* flowering factor *FLOWER LOCUS T* (*FT*), and the mutation of the intron region of this gene in barley led to early flowering [18,19]. *VRN1* and *VRN3* are flowering promotion factors, and long-term low temperatures can induce the expression of *VRN1* [8,10,20]. After vernalization, *VRN1* blocks the expression of the flowering inhibitor *VRN2* and promotes the expression of *VRN3* by binding with its promoter, thus inducing the flowering of winter wheat [19,21]. Photoperiod (*Ppd*) genes contain *Ppd-A1* (2A), *Ppd-B1* (2B), and *Ppd-D1* (2D), and out of these *Ppd-D1* has the largest impact on heading time in wheat [22,23,24].

The effects of vernalization genes *VRN1*, *VRN2*, and *VRN3* on plant heading time have been extensively studied. An insertion in the promoter region of recessive *VRN-A1* led to a later heading phenotype, whereas dominant allelic variants *VRN-A1*a, *VRN-A1*b, and *VRN-A1*c resulted in early heading due to a large fragment deletion of the promoter and first intron [25,26,27]. Additionally, 20–32 bp small deletions in the promoter region of *VRN-A1* and the variation of CArG box in *VRN1* led to early heading [11,28,29]. The allelic variations of *Vrn-B1* mainly occurred in intron1, and the *Vrn-B1b* allele caused spring growth habit in wheat [5,25,30]. Alleles of *Vrn-D*1 are mainly deletions or insertions in intron1 or the promoter region, and *Vrn-D1b* led to delayed heading time compared with *Vrn-D1a* [25,31]. *Vrn-D1b* has a 4235-bp deletion in intron1, accompanied by a single nucleotide mutation (G-A) in the promoter region [28]. The dominant *VRN3* allele carried a retroelement insertion in the promoter region, which resulted in the early flowering phenotype in wheat [18]. 

The copy number variation in plants is of great significance in the genetic regulation of plant growth and development [32,33,34,35]. It regulates many important agronomic traits such as plant height, stress resistance, and flowering time [31,33,36]. Of particular note, the copy number variation of *VRN-A1* and *Ppd-B1* is central to the regulation of wheat flowering time [31,37]. Exon 4 and exon 7 in *VRN-A1* contain a C/T double peak, suggesting that at least two copies were present. Further studies indicated that they were associated with flowering time [37,38]. The RILs with three copies of *VRN-A1* required a longer period of cold exposure to induce the transition to flowering than that of two copies [33]. The sequencing of 205 winter wheat varieties in China revealed that 22.9% of them contained one copy of *VRN-A1*, and the remaining 77.1% contained at least two copies [31].

In this study, we used gene mapping to identify a missense mutation in one copy of *VRN-A1*, which causes the late heading of wheat mutant *je0155*. Further investigation of *VRN* gene expression suggested that this mutation caused a lower transcription level of *VRN-A1*, which altered *VRN2* and *VRN3* expression and led to late heading in *je0155*. The identification of mutations in different copies of *VRN-A1* offers important insights for improving HD in wheat breeding. 

## 2. Results

### 2.1. A Single Recessive Gene Controlled Late Heading Phenotype of a Wheat Mutant 

**Figure 1:**
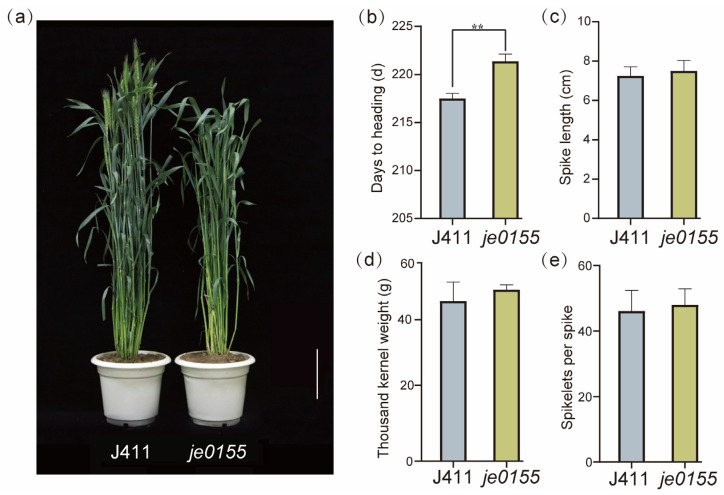
Comparison of phenotype and yield-related traits between WT and mutant: (**a**) Phenotypic comparison between WT (Jing411) and late heading mutant *je0155*. Plants were grown under field conditions and the picture was taken when the WT was heading. Bars = 10 cm; (**b**) Days to heading; (**c**) Spike length; (**d**) Thousand kernel weight; (**e**) Spikelets per spike. Values are averaged from five replicates. ** indicates significant differences by *t*-test at *p* < 0.01.

To explore and enrich the genetic regulation of heading date in wheat, we identified a late heading mutant *je0155* from our Jing411-derived mutant library. As shown in Figure 1a, the heading date of *je0155* was much later than that of wild-type Jing411 (J411). Further investigation suggested that the heading time of *je0155* was 3–4 days later than that of J411, presenting a statistically significant difference (Figure 1b). There appeared to be no difference in other agronomic traits such as spike length, thousand kernel weight, and spikelets number (Figure 1c–e). To map the genetic factors of heading date, we constructed an F_2_ population of 344 individuals derived from a cross between WT and mutant lines. The phenotypic analysis of F_2:3_ lines suggested that the ratio of early/semi-early heading and late heading lines fits a Mendelian model of 3:1 (Table S1), indicating that late heading is controlled by a single recessive gene.

### 2.2. A Primary Gene Affecting Heading Date Was Mapped on the Long Arm of Chromosome 5A

**Figure 2:**
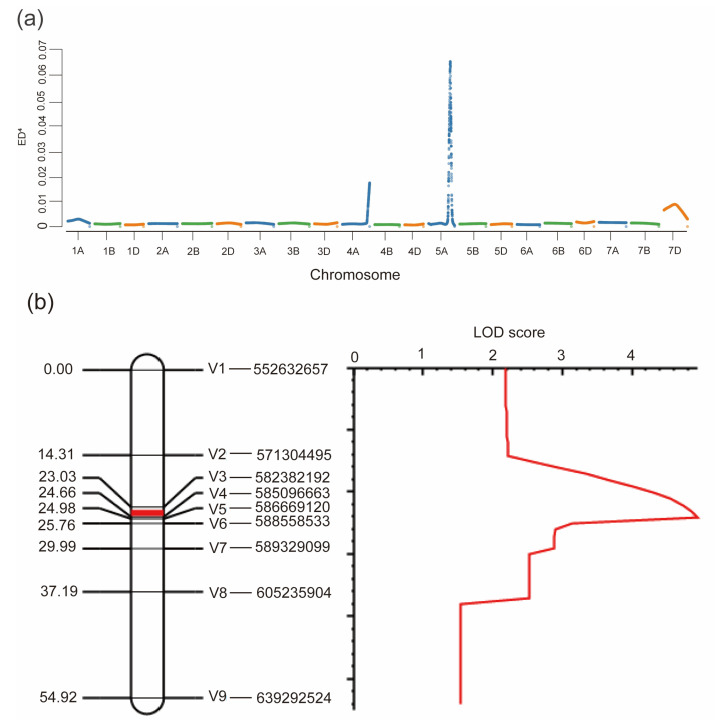
Mapping of the HD gene by BSA and genetic linkage analysis: (**a**) Distribution of ED^4^ values across all chromosomes. Higher ED^4^ values indicate a higher association effect with HD; (**b**) QTL analysis of HD on chromosome 5A. The logarithm of odds (LOD) threshold was set to 3.0, and a QTL located on a 1.63 cM of genetic linkage map between markers V3 and V4 was identified.

We performed Bulked Segregant Analysis (BSA) to map the gene for HD in the F_2_ population. Based on the F_2:3_ phenotype data, three early and three late heading bulks with roughly 30 individuals each, were used for BSA. Finally, a total of 22,705 SNPs were identified and used to perform further analysis. The Euclidean Distance (ED) value was calculated based on the reads and genotypes of bulks and their parents. The results detected a high peak region from 550 Mb to 650 Mb on chromosome 5A, and this region is speculated to be the gene locus associated with HD (Figure 2a). To validate the BSA-based mapping region, we successfully developed nine genome-specific KASP markers and genotyped the whole F_2_ population (Figure S1). We used QTL Icimapping analysis to create a linkage map covering this region on chromosome 5A. Based on the phenotypes of the F_2:3_ lines and the linkage map, we identified a major QTL with a LOD score of 4.9 and flanked by markers V3 and V4 with a genetic distance of 1.26 cM corresponding to a physical interval of 0.8 Mb according to the IWGSC RefSeq v2.1 (Figure 2b).

### 2.3. Mutations in Different Copies of VRN-A1 in je0155 

**Figure 3:**
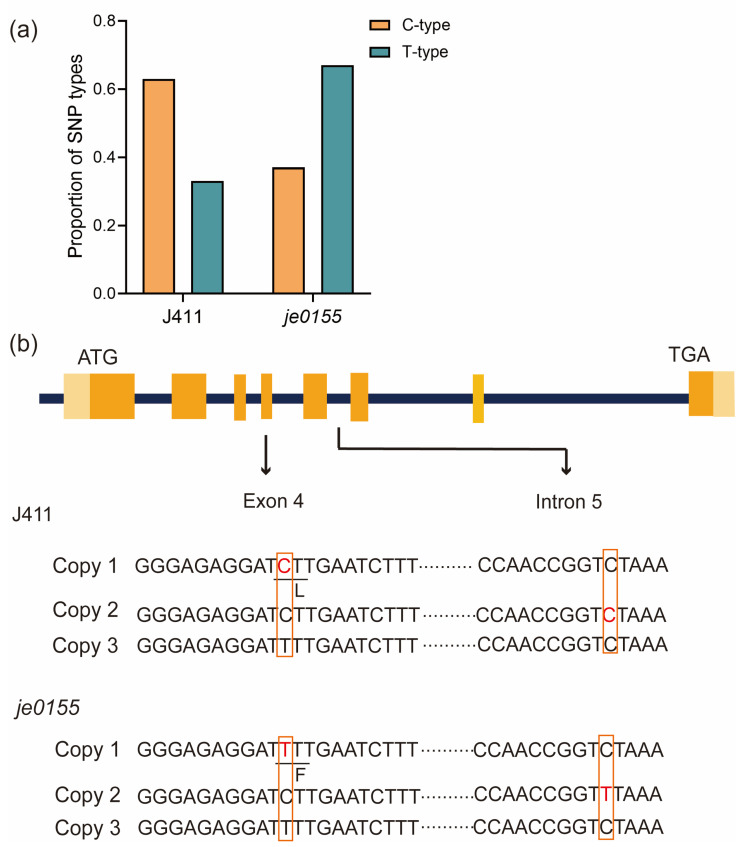
Copy number and mutation analysis of *VRN-A1* in *je0155*: (**a**) The ratio of C- and T-type alleles of exon 4 in WT and *je0155*; (**b**) Gene structure and sequence of three copies of *VRN-A1* with mutations in exon 4 and intron 5 of different copies of *VRN-A1* in *je0155*. The light orange boxes indicate the 5′ or 3′ UTR region, while the orange boxes indicate the exon regions. The red fonts represent the mutation site in J411 and *je0155*.

Since the vernalization gene *VRN-A1*, a key regulator of wheat heading and flowering [10], is located in the mapped region, we speculated that the *VRN-A1* would be the putative causal gene for heading date variation in *je0155*. After PCR amplification by genome-specific primers, we randomly selected clones of *VRN-A1* from WT and mutant lines for sequencing. Sanger sequencing of the individual clones suggested that two types of bases (C or T type) at the 10,606^th^ position in exon 4 were present in WT and *je0155*. The amounts of C- and T-type clones in WT were 46 and 26, respectively, with a ratio of 2:1, while the mutant fit a ratio of 1:2 with 26 and 50 clones (Figure 3a and Table S2). This indicates that WT and *je0155* have three copies of *VRN-A1*, and the mutant has a C to T mutation at the 10,606^th^ position in exon 4 in one of its copies (Figure 3b). This mutation results in an amino acid change from Leu (L) to Phe (F) in the K domain of *VRN-A1* protein, indicating that this missense variant is responsible for heading date variation in the mutant. Intriguingly, another copy of the C-type of exon 4 displayed a base mutation from C to T at the 11,141^st^ position of Intron-5 in *je0155*. Taken together, these two mutations in two copies of *VRN-A1* may contribute to the late heading phenotype in the mutant.

### 2.4. Mutation in VRN-A1 Affected Heading Time in the Population

**Figure 4:**
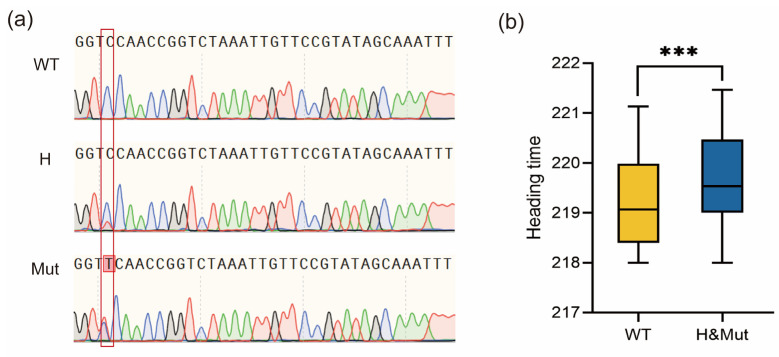
Development of PCR sequencing markers and the phenotypic analysis of the F_2:3_ population: (**a**) Sequences of different genotypes of mutations in intron 5 from the F_2_ population detected by Sanger sequencing markers. The red box represents different peaks detected by Sanger sequencing in the population; (**b**) Heading time comparison of F_2:3_ lines genotyped by PCR sequencing markers. WT, H, and Mut indicate homozygous WT, heterozygous, and homozygous mutant genotypes, respectively. *** indicates significant differences at 0.001 level by *t*-test.

To verify the effects of *VRN-A1* mutations on heading date, we developed a molecular marker for the intron 5 mutation site through PCR sequencing. Due to containing three copies of the *VRN-A1* gene, the WT showed a single peak of C at the mutation site, while the mutant showed overlapping peaks of C and T, which is consistent with the peaks of heterozygous plants (Figure 4a). Using this marker, we analyzed the genotypes of the F_2_ population. By combining genotypic and phenotypic data from this population, we determined that the heading date of plants with mutated and heterozygous genotypes was significantly later than that of WT plants (Figure 4b). Due to the allelic variation in different copies of *VRN-A1*, we could not use molecular markers to distinguish the mutation in exon 4 between WT and *je0155*. However, since different copies of *VRN-A1* are tightly linked, the genotypes detected by markers of intron 5 also reflect genotypes in exon 4. These results indicate that the mutations in *VRN-A1* putatively contributed to the later heading phenotype in *je0155*.

### 2.5. Expression Pattern of VRN1, VRN2, and VRN3 Genes

**Figure 5:**
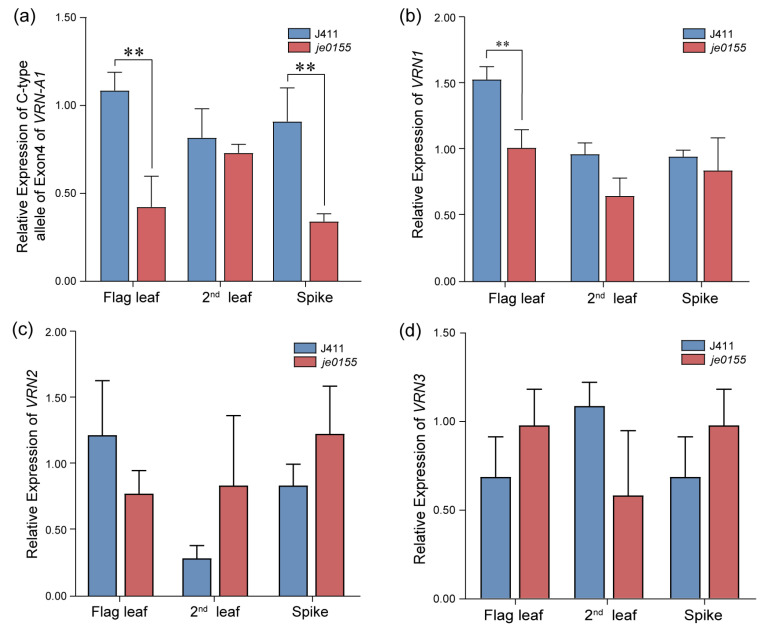
Real-time qPCR of vernalization genes in WT and *je0155*. Values are means ± SD from five biological replicates. Second leaf indicates the second leaf below the spike: (**a**) Expression of C-type allele of exon 4 of *VRN-A1* in the flag leaf, the second leaf, and the young spike of WT and *je0155*; (**b**–**d**) Relative expression of *VRN1* (**b**), *VRN2* (**c**), and *VRN3* (**d**). ** represents a significant difference at 0.01 level by *t*-test.

To analyze expression changes of vernalization genes between WT and mutant lines, we conducted an RT-qPCR for the C- and T-type alleles of exon 4 of *VRN-A1* and the *VRN* genes in the flag leaf, the second leaf, and the spikes at the early heading stage. In the flag leaf and spike of *je0155*, the expression level of the C-type allele of exon 4 of *VRN-A1* was significantly decreased compared with that of WT (Figure 5a). There was no significant difference between the expression levels of the *VRN-A1* T-type allele in the WT and mutant lines (Figure S2). Additionally, the expression level of *VRN1* was significantly decreased in the flag leaf in *je0155* compared to WT (Figure 5b). In contrast, the expression of *VRN-A1* was similar between the spikes of the WT and mutant lines. Generally, the expression of *VRN2* and *VRN3* genes showed no significant difference between WT and mutant in different tissues. Notably, the expression of *VRN2* was slightly increased, while that of *VRN3* was decreased in the second leaf of *je0155* compared to WT (Figure 5c,d).

## 3. Discussion

The heading date is an important factor in determining the ability of different wheat varieties to adapt to their local climate conditions and environmental stresses [39,40]. The time of heading directly influences grain yield in wheat, making it an important characteristic to consider when breeding new varieties [41]. The development of novel genetic resources and the identification of the key genes associated with heading date are needed to provide important cues for the modification of HD in wheat breeding. In this study, we identified new germplasm, EMS-induced mutant *je0155*, with HD variations of 3–4 days. By combining BSA with genetic mapping, a stable QTL was identified on a physical interval of 0.8 Mb on chromosome 5A (Figure 2a,b), and this region contained the *VRN-A1* gene. Further gene cloning and sequencing revealed that the *VRN-A1* gene has three copies in both WT and mutant lines. These results are consistent with a previous study that found a high percentage of Chinese wheat varieties contained three copies of *VRN-A1* [32]. It has been reported that the distinct copies of *VRN-A1* showed either a C or T in exon 4 [37] and these different alleles are central to the regulation of flowering time [42]. In our study, one copy of the C-type of exon 4 in WT was mutated into a T-type in *je0155*, which resulted in a conserved amino acid change from Leucine to phenylalanine. A previous study suggested that the T-type allele of *VRN-A1* results in a deleterious substitution and that the new encoded protein is less active than that of the C-type. The expression of the C-type allele was also found to affect flowering time more strongly than that of the T-type allele [42]. It is reasonable to speculate that one copy of the C-type allele changed to a T-type in the mutant, resulting in the delayed HD phenotype. Similarly, the high frequency of the C-type allele in the Buster-like cultivar resulted in an earlier flowering phenotype, while the high frequency of the T-type allele in the Charger-like cultivar triggered a later flowering phenotype [42]. Therefore, we have determined that the mutation of C to T in exon 4 is likely responsible for late HD in *je0155*.

However, we could not exclude the possibility that the mutation in intron 5 of another copy of the C-type allele may have also contributed to a postponed HD in the mutant. Previous studies have suggested that both the large fragment deletion in the first intron of *VRN-A1* and the C to A mutation in the GArG box of the *Vrn-D1b* promoter resulted in delayed heading [28,29,30]. Moreover, a mutation in the non-coding region of *VRN3* was found to cause HD variations in an array of winter wheat varieties in China [43]. In our study, genotype and phenotype analysis of the segregation population suggested that the mutation in *VRN-A1* contributed to HD variations (Figure 3 and Figure 4). Since both identified mutations in the duplicates of *VRN-A1* were tightly linked, we could not conclude which mutation contributed to delayed HD. 

The mutation in exon 4 may affect the expression of *VRN-A1*, thereby influencing the HD in *je0155*. Transcript level analysis suggested that the expression of the C-type allele in the flag leaf and young spikes of the mutant was around half of that in WT, while the expression of the T-type allele showed no significant difference between the WT and mutant (Figure S2). Consistently, the early flowering wheat cultivar Buster expressed a higher amount of C-type alleles than the later flowering wheat cultivar Charger [42]. It is well documented that as the central regulator of flowering, *VRN-1* represses the expression of *VRN2* while increasing *VRN3* expression to promote flowering after vernalization in wheat [11,18]. Accordingly, the lower expression of C-type *VRN-A1* resulted in a relatively higher expression of *VRN2* and a slightly lower expression of *VRN3* (Figure 5), which led to late heading in the mutant. 

In conclusion, this study mapped the gene responsible for HD variation in the wheat mutant *je0155* to a 0.8 Mb region of chromosome 5A by BSA and genetic linkage analysis. Through gene cloning and sequencing analysis, we found that a missense mutation in exon 4 of one copy of *VRN-A1* contributes to late heading in the mutant. This mutation caused a lower expression of *VRN-A1*, resulting in an increase in *VRN2* and a slight decrease in *VRN3* transcription, thereby leading to the late heading phenotype in the mutant. The allelic mutations of different copies of *VRN-A1* identified in this study provide a novel genetic resource for the modification of HD in wheat breeding. By developing our understanding of the genetic regulation of HD, we hope to contribute to the creation of more adaptable wheat varieties.

## 4. Materials and Methods

### 4.1. Plant Materials and Phenotypic Analysis

The late heading mutant (*je0155*) was obtained by using Ethyl methane sulfonate (EMS) to mutagenize the wheat variety Jing411 (J411). The mutant *je0155* was crossed with J411 to produce an F_2_ population. For genotype detection, 344 F_2_ plants were sown to create F_2:3_ lines, each of which consisted of 30 plants in two rows. The F_2:3_ population (344 lines) and parent lines were sown at the Changping station of the Institution of Crop Science of the Chinese Academy of Agriculture Sciences (Beijing, China). The heading time of each plant was recorded when two-thirds of the spikes had emerged. At least 15 plants from each line were used for phenotypic investigation. 

### 4.2. Genomic DNA Extraction

Fresh leaf samples at the seedling stage were processed for DNA extraction. First, the leaves were frozen and ground to a uniform powder using a Vibration Mill Type MM301 (Restsch GmbH, Germany) and completely mixed with 600 μL DNA extraction buffer. Samples were incubated at 65 °C for 1 h, then mixed with 200 μL 5 mol∙L^−1^ KAc and centrifuged. A 300 μL aliquot of the supernatant was collected from each sample, and the resulting DNA was precipitated by isopropanol and washed twice with 70% ethanol. A NanoDrop ND-2000 spectrophotometer (Thermo Scientific) was used to measure the quality and quantity of DNA, and the final concentration was normalized to 60 ng∙μL^−1^. 

### 4.3. Bulked Segregant Analysis (BSA)

Based on the heading time data of the F_2:3_ families, the 93 early-heading plants and 75 late-heading plants were selected to construct three early-heading mixed pools and three late-heading mixed pools. A total of 500 ng of DNA was taken from each sample and mixed into the corresponding pool. Simultaneously, 8 individual plants of J411 and *je0155* were selected to construct the parental pool. In this study, the Illumina HiSeq X high-throughput sequencing platform was used to sequence the whole exome of six progenitor mixed pools and two parent pools. Exome capture sequencing and analysis of the original sequencing data (Tcuni Technologies, Chengdu, China) were conducted as previously reported [44]. After sequencing, the raw data was filtered and all low-quality reads were removed, resulting in a total of 170.01 Gb of clean data. The Burrows–Wheeler Aligner (BWA) software was used to align the filtered data to the Chinese Spring IWGSC v1.1 reference genome. After the data was aligned, SAMtools was used to convert the matched files into BAM files, Biobambam2 software was used to remove duplicate reads generated by PCR, and BCFtools was used to detect SNPs to obtain VCF files. After analysis, 536,272 SNPs were obtained. We then used Euclidean Distance (ED) algorithm to calculate the relative allelic frequencies between two bulks, which reflects the correlation between markers and traits of interest [45]. To eliminate background noise, the ED value was fitted using a Loess curve, and the region with a high peak of ED was speculated as a candidate region.

### 4.4. Development of Kompetitive Allele Specific PCR (KASP) Markers

Based on BSA data, the SNPs between two parent lines on candidate chromosomal region were selected and converted to Kompetitive Allele Specific PCR (KASP) markers by using the online primer design pipeline PolyMarker (http://polymarker.tgac.ac.uk/) (accessed on 29 July 2021). The parent plants were used to detect the specificity of KASP primers. The PCR reaction was performed using a CFX 96 Real-Time System (Bio Rad, Hercules, CA, USA). A 5μL reaction system consisted of 2.5 μL KASP master mixture (LGC Genomics, Middlesex, UK), 2.4 μL 60 ng∙μL^−1^ DNA, 0.04 μL 50 mM Mg^2+^, and 0.06 μL primer mix (primer A (100 μM):primer B (100 μM):primer R (100 μM):ddH_2_O = 12:12:30:46). A PCR amplification procedure was conducted for: 95 °C for 15 min, followed by 10 cycles of touch-down (95 °C for 20 s; touch-down at 65 °C initially and decreasing by 0.5 °C per cycle for 30 s), then followed by 30 additional cycles of annealing (95 °C for 10 s; 57 °C for 60 s). All KASP primers were described in Table S3.

### 4.5. Construction of a Genetic Linkage Map

Based on the genotypes detected by KASP markers in the whole F_2_ population, a linkage map was constructed using the MAP function of IciMapping version 4.2. Recombination frequency was converted into centimorgan (cM) distances with the Kosambi map function. The estimates of recombination fraction pairwise between all markers are listed in Table S4. Based on the linkage map and heading date from F_2:3_ lines, QTL mapping was conducted using the inclusive composite interval mapping (ICIM) analysis. A value of phenotypic variance (PVE) explained by an individual QTL was determined using ICIM. Significant QTLs were identified by permutation tests with 1000 times at an error threshold of 0.05 and a logarithm of odds (LOD) threshold of 3.0.

### 4.6. Identification of VRN-A1

Four pairs of primers were designed to amplify the whole length of *VRN-A1*. To sequence *VRN-A1*, the whole gene was divided into two segments prior to amplification. PCRs were performed using 2× Phanta Flash Master Mix Dye Plus (Vazyme) with the following procedure: 98 °C for 30 s, followed by 35 cycles of annealing (98 °C for 10 s, 60–65 °C for 5 s and 72 °C for 1 min 30 s), and 72 °C for 1 min. Then, the PCR product was cloned using the pEASY^®^-Blunt zero-cloning vector (Transgen) at 37 °C, 1:7 for 30 min. After transformation into receptive cells, the universal primer M13 was used for detection, and the positive clones were cultured overnight. All primers used are described in Table S3.

### 4.7. Quantitative Real-Time PCR Analysis

To quantify the relative expression of *VRN-A1* genes, the spikes, flag leaves, and the second leaves of J411 and *je0155* were sampled at the early heading stage. Total RNA from each part was isolated using a TransZol Up Plus RNA Kit (TransGen). By using DNase I (Takara) and an RNA purification kit (Tiangen), all DNA contamination in the extracted RNA was eliminated. A TransScript First-Strand cDNA Synthesis SuperMix kit (TransGen) was used to synthesize the first strand cDNA from the extracted RNA. A qRT–PCR was conducted using the ChamQ Universal SYBR qPCR Master Mix (Vazyme) and CFX 96 Real-TimeSystem (Bio-Rad, Hercules, CA, USA). This experiment was performed with five independent biological replicates and three technical repeats. For expression analysis of *VRN1*, *VRN2*, and *VRN3* genes, primers described in previous studies were used [42,46]. The internal control gene *ACTIN* was used to normalize the expression data. Relative expression levels were determined using the 2^−ΔΔCT^ method.

## Data Availability

Not applicable.

## Supplementary Materials

The following supporting information can be downloaded at: https://www.mdpi.com/article/10.3390/ijms24055008/s1.
