# Supplementary material for: Gene Mapping and Identification of a Missense Mutation in One Copy of VRN-A1 Affects Heading Date Variation in Wheat
Source: Int J Mol Sci. 2023 Mar 5;24(5):5008. doi: 10.3390/ijms24055008 (PMC10003625; doi:10.3390/ijms24055008)
Supplement: Supplementary file 1 [file ijms-24-05008-s001.zip › ijms-2124191-Figures S1 and S2.pdf]

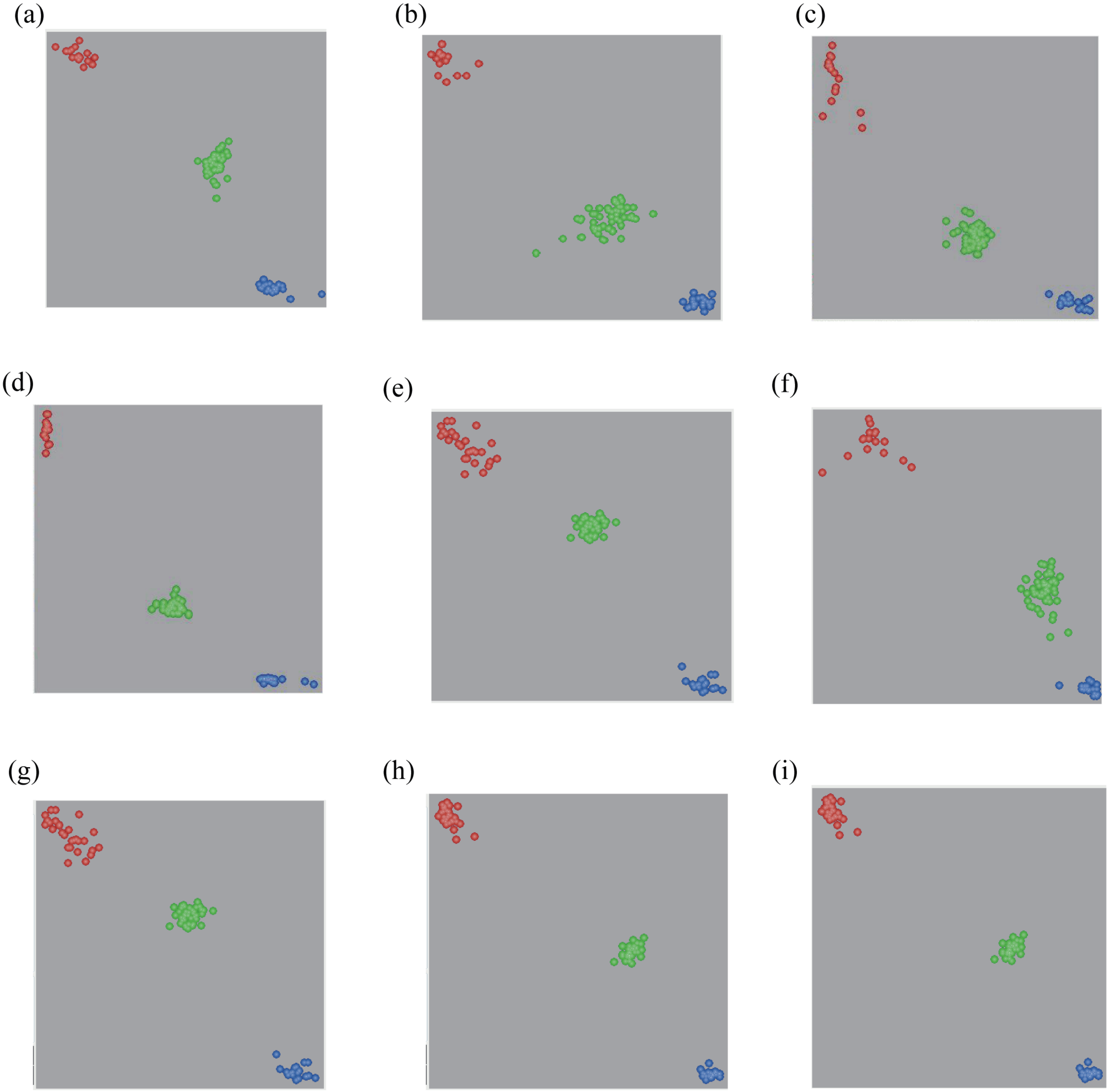

Figure S1. Genotypes detected by KASP markers. (a) V1, (b) V2, V3(c), V4(d), V5 (e), V6(f), V7 (g), V8 (h) and V9 (i). The blue and red dots represent the wild type and mutant alleles, respectively, while the green dots represent the heterozygous alleles.

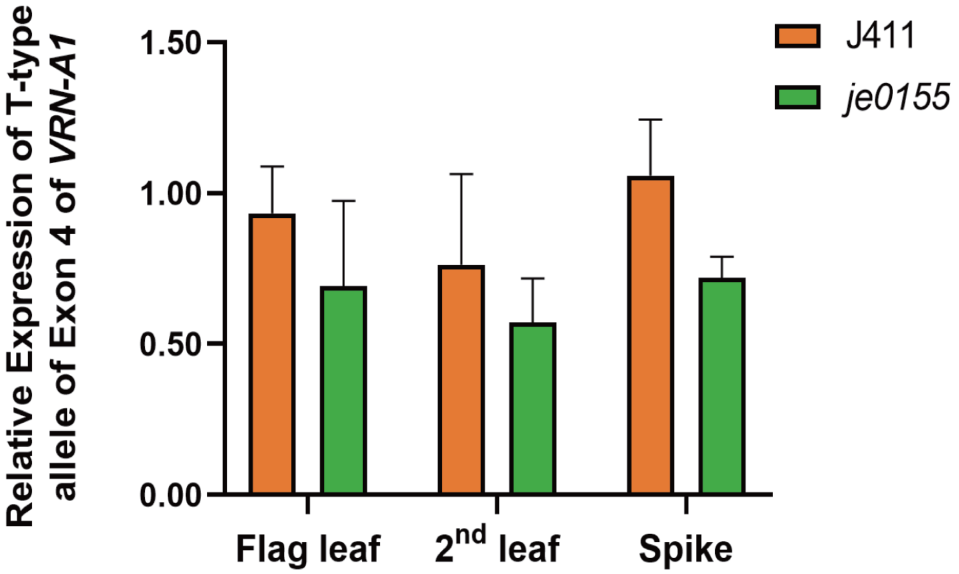

Figure S2. Expression of T-type of exon 4 of *VRN-A1* in the flag leaf, the second leaf, and the young spike.
